# Effects of Cold Rolling Reduction on Microstructure, Thickness, Adhesive Force of Al-Si Coating and on Bending Toughness of Al-Si Coated Press-Hardened Steel

**DOI:** 10.3390/ma16010004

**Published:** 2022-12-20

**Authors:** Xue Feng, Xianlei Hu, Xianghua Liu

**Affiliations:** 1 School of Materials Science and Engineering, Northeastern University, Shenyang 110819, China; 2 Suzhou Dongbaohaixing Metal Material Science and Technology Co., Ltd., Suzhou 215625, China

**Keywords:** cold rolling, AS60/60 coating, austenitization, microstructure, thickness, adhesive force, bending toughness

## Abstract

Al-Si coated press-hardened steel (PHS) is widely used along with the development of light-weight vehicles, and the tailor-rolled blank parts based on Al-Si coated PHS have attracted much attention. The preparation process includes cold rolling, austenitization, hot-stamping, and quenching. The most widely used AS60/60 coating will change after cold rolling and austenitization, which has been little-studied. Herein, the effects of cold rolling reduction on the microstructure, thickness, adhesive force of AS60/60 coating and on bending toughness of AS60/60 coated PHS were studied. As the cold rolling reduction ratio increased from 0% to 50%, the coatings were gradually thinned, but the overall continuity was unchanged. When the reduction ratio was 40% or above, rapid diffusion channels were formed. The adhesive force of coatings was 21.50–22.15 MPa. After austenitization, the coating thickness gradually decreased as the cold rolling reduction ratio rose from 0% to 50%, but the structure and overall continuity were both unchanged, and the adhesive force was 21.60–22.40 MPa. The rapid diffusion channels promoted the transition from brittle Fe2Al5 to tough FeAl during austenitization, leading to a rapid increment in bending toughness after Al-Si coated PHS was quenched. When the reduction ratio was 50%, the bending angle was improved by 23%.

## 1. Introduction

To address the greenhouse effect and improve global competitiveness in the automobile industry, all countries have put forward stricter requirements on light-weight vehicles. Reportedly, when the weight of a vehicle is lightened by 10%, the oil consumption can be reduced by 6–8%, and the exhaust gas emission will also be decreased [1,2]. The light weight of vehicles is achieved by choosing low-density materials, improving material strength, or optimizing part structures to decrease weights, while ensuring the collision safety of vehicles. press-hardened steel (PHS) is widely used in light-weight vehicles owing to its superhigh strength, low forming resilience, and process stabilization [3,4,5,6,7,8].

22MnB5, the commonly-used PHS, is formed by heating steel blanks to 930–960 °C and preserving there for 3–6 min to induce austenitization. Then, the steel is immediately transferred to a hot stamping die, where the steel is stamped at high temperature and rapidly cooled at the rate above 30 °C/s to form martensite [9,10,11,12]. When uncoated steel blanks are austenitized in a heating furnace and then transferred to the die, oxidation or decarbonization will occur on the surface. During the manufacturing, the iron oxide skins falling into the die should be periodically removed [13], and the formed parts should be shot-peened to discard the oxide layer, leading to lower production efficiency and the deformation of thin specified parts [5]. An Al-Si layer coated on the surface of steel blanks can avoid the formation of iron oxide skins, and achieve high corrosion resistance after hot stamping [14,15]. With the Al-Si coating technology proposed and applied by Arcelor Mittal, the steel blanks leaving a continuous annealing furnace are hot-dipped at 660–680 °C. The typical composition of the coating is 87Al-10Si-3Fe, the coating thickness is 20–33 μm, and a thin Al_2_O_3_ layer exists on the coating surface [16,17]. During austenitization, the Al and Si in the coating and the Fe in the steel substrate mutually diffuse, forming a Fe-Al intermetallic compound with a melting point far above the austenitization temperature. Moreover, a very thin Al_2_O_3_ layer is formed on the coating surface, which effectively prevents the substrate from oxidation. However, the bending toughness of Al-Si-coated PHS is reduced by more than 20% compared with the PHS without coating after hot-stamping [4], which will reduce the collision safety of vehicles.

Tailor-rolled blanks are blanks with continuous variation in length-direction thickness. The thickness and mechanical properties in different zones of a steel blank can be designed according to the bearing status of different parts, which will effectively lighten the parts and improve collision safety. The combination of PHS and tailor-rolled blanks will further reduce the weights of parts. In a typical preparation process of hot stamping tailor-rolled blanks, the isopachous materials are flexibly cold-rolled, straightened and cut, and the roll-hardened blank materials are blanked and directly hot-stamped. When the Al-Si coated PHS is flexibly rolled, the coatings are thinned and fragmented accordingly, so the phase composition and phase proportions of the intermetallic compounds formed in the subsequent austenitization may differ. For this reason, studying the effects of cold rolling reduction ratio on the cold-rolled and austenitized Al-Si coatings is very important.

Some researchers have studied the effects of Si concentration on the hot-dipping of Al-Si coatings. B. Lemmens, H. Springer et al., thought that during Al-0wt.% Si hot-dipping, the coatings contained a very thick tongue-like η-phase compound (Fe_2_Al_5_) at the steel substrate side, and a θ-phase compound (Fe_4_Al_13_) at the Al side. The coatings during Al-10wt.% Si hot-dipping contained very thin η phase, θ phase, and τ_5_ phase (Fe_2_SiAl_7_) [18,19,20]. W.J. Qiang thought the thickness of the interfacial η-phase compound is reduced because the interfacial compound Fe_2_Al_5_ in the pure Al coating only had 70% atom saturation. In the lattices, numerous cavities appear at the c-axis, and Si atoms fill in the cavities of Fe_2_Al_5_, so the Al atoms cannot pass through the cavities to diffuse to the boron steel substrate, inhibiting the growth of Fe_2_Al_5_ [21]. W.J. Cheng thought the thickness of the interface compound layer is significantly shortened when the Si content rises below 10%, but is not changed obviously when the Si content rises above 10% [22]. The phase transformation and structure evolution during austenitization of Al-Si coatings were also studied [23,24,25,26]. M. Windmann et al. thought Fe diffusion from the steel substrate to the Al-based coating was predominant within the first 2 min when Al-10% Si coatings were austenitized at 920 °C. After 2 min. Al was diffused from the Al-based coating to the steel substrate, so the Al-rich intermetallic compounds (Fe_4_Al_13_, Fe_2_Al_5_) were transformed to Fe-rich FeAl intermetallic compounds. The Al diffusion to the steel substrate helped form an Al-rich α-Fe layer at the substrate side, and the layer thickness was enlarged with the rise of austenitization temperature and the prolonging of heat preservation time [17]. The anti-deformation ability of Al-Si coatings was also studied. H.J. Wei et al. believed raising the aqueduct temperature can decrease the likelihood of cold cracking in Al-10% Si coatings [27]. M.X. Huang et al. thought brittle thin intermetallic compounds and a ferrite layer were formed during austenitization of thin Al-Si coatings, so the coating cracks were short, and the SIF at the crack tip was low, thereby improving the bending strength [28].

So far, there is little research on the effects of cold rolling reduction ratio on Al-Si coatings. In this study, the effects of reduction ratio on cold-rolled and austenitized Al-Si coatings were investigated. The correlations of cold rolling reduction ratio with the structure evolution, thickness, and adhesive force of coatings and the bending toughness of PHS were elaborated. The findings will underlie the development of Al-Si-coated PHS tailor-rolled blanks.

## 2. Materials and Methods

### 2.1. Materials and Cold Rolling

The hot-dip Al-Si coated PHS used here was featured by standard AS60/60 coating and a 22MnB5 substrate. The chemical composition of 22MnB5 was detected using optical emission sparking spectroscopy (Table 1).

**Figure 1:**
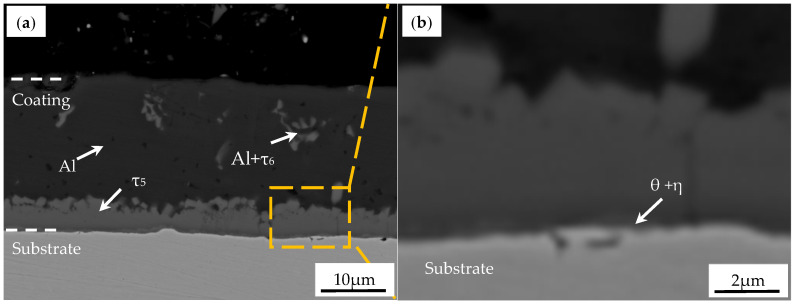
Electron probe microanalyzer (EPMA) micrographs of the interface between the AS60/60 coating and the steel substrate in the hot-dipped condition: (**a**) AS60/60 coating; (**b**) micrograph of the area marked by the yellow rectangle in (**a**) at higher magnification.

The AS60/60 coated PHS was 1.8 mm thick, and the coating layer was 27 μm thick. The microstructures of the AS60/60 coated PHS are shown in Figure 1. The AS60/60 coatings were structurally continuous without cracks or pores. The coatings were stratified with clear boundary. No Fe_2_Al_5_-phase tongue-like structure was found at the interface between the coating and the steel substrate. This was because Si atoms added into the liquid-state Al filled in the c-axial cavities in the orthorhombic Fe_2_Al_5_ phase, which blocked Al atom diffusion and inhibited Fe_2_Al_5_ growth to the substrate direction. The high-power electron probe shows that a thin layer of interface compounds exists in between the τ_5_ phase and the substrate. Reportedly, the η phase near the steel substrate is Fe_2_Al_5_, and the θ phase near τ_5_ is Fe_4_Al_13_ [18,20,26].

The coatings at different positions were processed with energy dispersive spectroscope (EDS) in Table 2. The Al-Fe-Si ternary phase diagram shows ten stable intermetallic compounds: Fe_3_Si_3_Al_2_ (τ_1_ or τ_9_), FeSiAl_3_ (τ_2_ or γ), FeSiAl_2_ (τ_3_), FeSi_2_Al_3_ (τ_4_ or δ), Fe_2_SiAl_7_ (τ_5_ or α), FeSiAl_4_ (τ_6_ or β), Fe_2_Si_3_Al_3_ (τ_7_), Fe_3_Si_4_Al_2_ (τ_8_), Fe_4_Si_3_Al_9_ (τ_10_), and Fe_2_SiAl_5_ (τ_11_) [29]. Table 2 and phase diagram analysis demonstrate the hot-dip coatings are composed, from the surface to the substrate, of an Al layer (Al phase + Si phase + τ_6_ phase) and an intermetallic compound layer (IMC layer, τ_5_ phase + θ phase + η phase).

The hot-dip AS60/60-coated PHS was cut into rectangular specimens (150 × 100 mm) and then rolled on a four-roller cool rolling machine at the reduction ratio of 0% (defined as the original specimens), 10%, 20%, 30%, 40%, or 50%. In the austenitization process, the cold-rolled specimens from the above step were heated at 8 °C/s in an MXQ1200-30 box-typed air furnace to 930 °C and preserved there for 5 min. Then, the specimens were taken out, immediately put into the blank die of a hot-stamping machine, and cooled at the rate above 40 °C/s to room temperature.

### 2.2. Microstructure Characterization and Property Test

The Al-Si coated hot-stamping specimens processed at different reduction ratios were sampled in the size of 10 × 8 mm through electrospark processing. The samples were corroded with a 4% nitric acid alcohol solution and observed under optical microscopy (OM). Microstructures were observed under a JEOL JXA-8530F electron probe microanalyzer (EPMA) at the accelerating voltage of 20.0 kV. The phase compositions of the coated structures were analyzed using the EDS carried in the EPMA. The element distributions in the coated sections were investigated using the X-ray line scanner and X-ray map scanner carried in the EPMA. The thickness of the Al-Si coatings processed at different cold rolling reduction ratios was detected using an OLYMPUS BX53M optical microscope.

The lap shear strength of the coatings was measured through tensile tests to characterize the effect on the adhesive force of coatings. The specimen sizes and GM-approved high strength impact resistant full spectrum adhesive DP460 specified in standard GM15200 were adopted. The typical glue layer thickness of 0.2 mm was achieved by inserting spacing wires in parallel to the tensile force direction. Tensile tests were conducted on an SANS CMT5105 microcomputer-controlled electronic universal testing machine at the tensile speed of 10 mm/min as per the GMW15200 standard. Three specimens at each reduction ratio were tested and the data were averaged.

The specimens quench-cooled to room temperature were put into an MXQ1200-30 box-typed air furnace and preserved at 180 °C for 20 min. After that, the specimens were taken out from the furnace and air-cooled. The cooled specimens were electrosparked into the size of 60 × 60 mm. Bending tests were carried out as per the VDA238-100 standard. Three specimens at each reduction ratio were tested and the data were averaged.

## 3. Results and Discussion

### 3.1. Microstructure, Thickness and Adhesive Force of Cold-Rolled Coating

**Figure 2:**
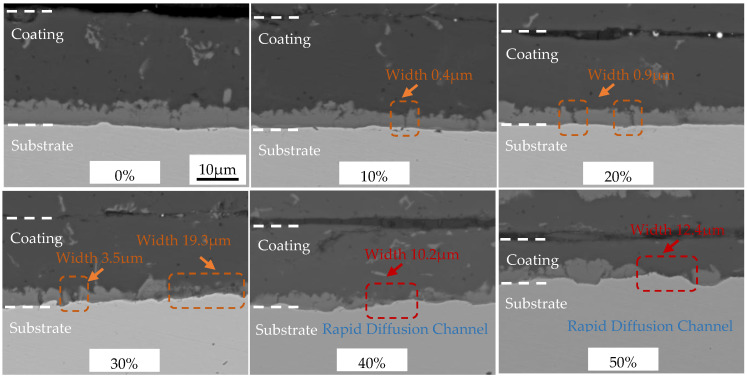
EPMA micrographs of AS60/60 coating at different reduction ratios.

The microstructures of the AS60/60 coatings at different cold rolling reduction ratios were illustrated in Figure 2. The AS60/60 coatings at different reduction ratios were continuous on the whole, without any cracking, peeling or shedding. As the reduction ratio increased, the Al layer was gradually thinned, but was still continuous without cracking. The IMC layer thickness was not related to the reduction ratio and was basically unchanged (about 5 μm). Nevertheless, the status of the IMC layer changed, as it was fragmented and cracked. The IMC layer of the hot-dipped raw materials was uniform and continuous. At the reduction ratio of 10%, the IMC layer cracked throughout, and the cracks were perpendicular to the substrate. When the reduction ratio increased, the crack density rose and the cracks transversally expanded and thereby widened. At the reduction ratio of 30%, the crack width was enlarged from 0.4 to 3.5 μm, and a regional crack in width of 19.3 μm appeared. When the reduction ratio was above 40%, the crack density in the IMC layer was lowered, and the regional crack was filled with the Al layer. At the same time, the θ + η interfacial compound layer at the filling site was absent. Thus, direct contact between the Al layer and the substrate will help with the rapid Al diffusion to the substrate and the rapid Fe diffusion to the Al layer during the subsequent austenitization, forming rapid diffusion channels.

**Figure 3:**
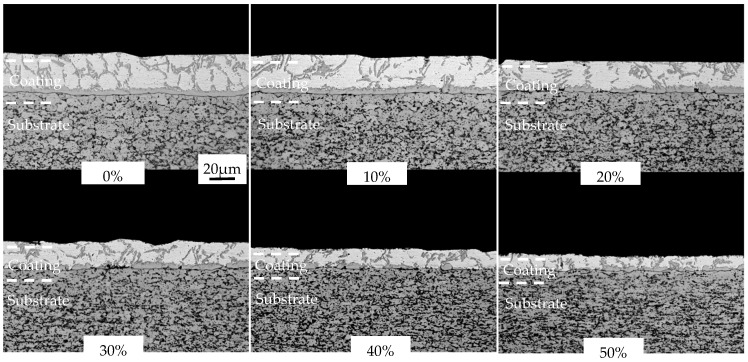
Optical microscopy (OM) micrographs of AS60/60 coating at different reduction ratios.

**Figure 4:**
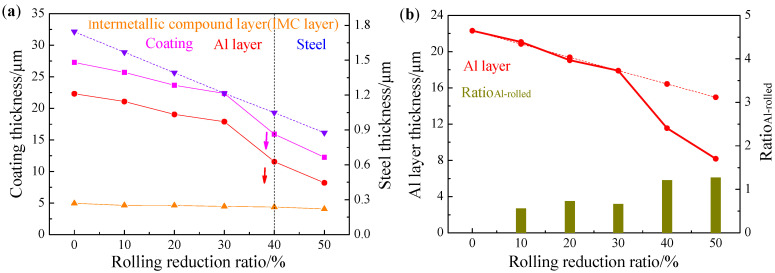
(**a**) AS60/60 coating thickness and steel substrate thickness at different reduction ratios; (**b**) Al layer thickness and Ratio_Al-rolled_ at different reduction ratios.

The AS60/60 coatings at different cold rolling reduction ratios were displayed in Figure 3, and the corresponding coating thickness and steel substrate thickness were shown in Figure 4. The original AS60/60 coatings were 27.28 μm thick, in which the Al layer was 22.32 μm thick and the IMC layer was 4.96 μm thick. As the reduction ratio increased, the coating thickness was shortened in a nearly linear way, and the thickness shortening rate was accelerated when the reduction ratio was ≥40%. The changing trend of the Al layer thickness was consistent with that of the coating thickness, but the IMC layer thickness was maintained at 4–5 μm.

When the cold rolling reduction ratio was ≤30%, the ratio of Al layer deformation rate to the cold rolling reduction ratio, ratio_Al-rolled_, was 0.65 on average (Figure 4b). When the cold rolling reduction ratio was ≥40%, the mean ratio_Al-rolled_ was 1.24, which is consistent with the phenomenon above that the Al layer filled in the IMC layer. In other words, when the cold rolling reduction ratio was ≥40%, the Al layer filled in the cracks in the fragmented IMC layer, leading to an accelerated decrease of Al layer thickness. When the cold rolling reduction ratio increased, the IMC layer was significantly fragmented, and the Al filling rate and the filling effect were improved.

**Figure 5:**
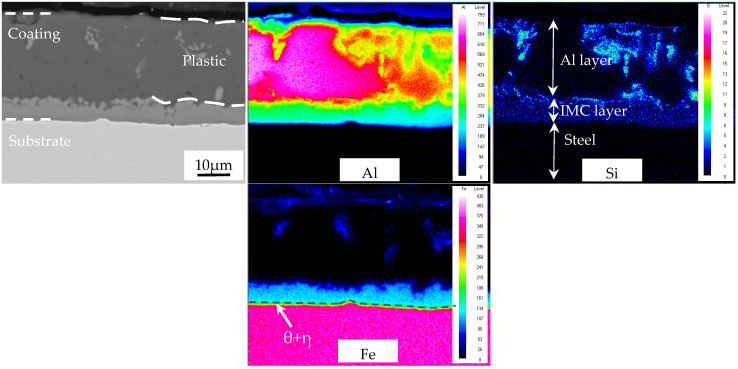
EDS map scan of interface between the AS60/60 coating and the steel substrate at 0% reduction ratio.

The AS60/60 coating was map-scanned at the reduction ratio 0% in Figure 5. The richest element in the Al layer was Al, but the Al concentration varied largely, and was complementary with the Si concentration distribution. This was mainly because in a eutectoid reaction (L→Al + Si), Fe was scattered in an island-like way mainly due to the crystallization reaction (L→τ_6_). In the IMC layer, Al, Fe, and Si were all distributed uniformly. At the substrate side of the IMC layer was an interface compound layer of thickness of ~1 μm.

**Figure 6:**
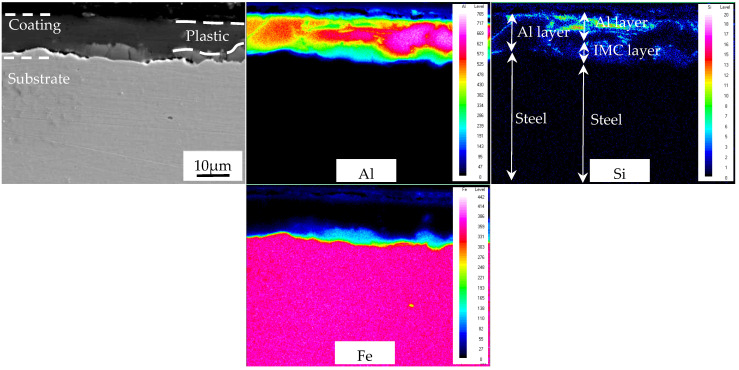
EDS map scan of interface between the AS60/60 coating and the steel substrate at 50% reduction ratio.

The Al-filled zone of the AS60/60 coatings was map-scanned at the reduction ratio 50% (Figure 6). The map scan of the AS60/60 coatings was divided into two types: “Al layer, IMC layer, steel substrate”, and “Al layer, steel substrate”. The map scan more visually showed the concentration distributions of various elements at the rapid diffusion channels. The map scan results at “Al layer, IMC layer, steel substrate” are consistent with the map scan results of the 0% reduction ratio. In the IMC layer, there is a buffer zone (about 5 μm thick) composed of Al and Fe concentration distributions as well as an Si enrichment zone. At the “Al layer, steel substrate” rapid diffusion channels, there is no buffer zone composed of Al and Fe concentration distributions, or the Si enrichment zone.

**Figure 7:**
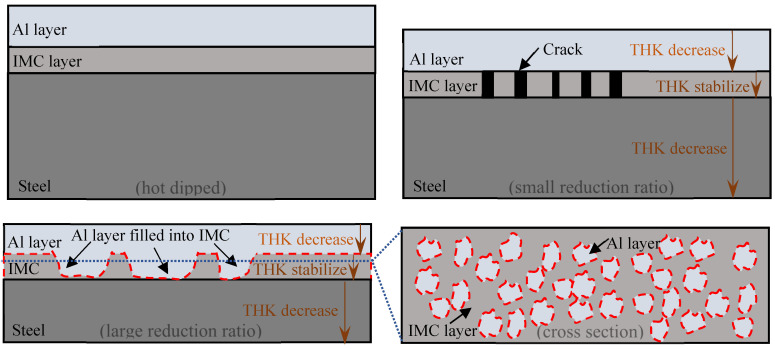
Deformation of Al-Si coating during cold rolling.

The hardness of the AS60/60 coatings was detected, and the hardness of the Al layer and the IMC layer was about 70 and 600 HV, respectively. The hardness of the B steel substrate increased with the rise of cold rolling reduction ratio. At the reduction ratios of 0–50%, the substrate hardness was 175, 206, 227, 242, 258, 267, 274 HV, respectively. During the cold rolling, the Al layer, the IMC layer, and the B steel substrate were all deformed; the Al layer and the substrate both with high ductility were gradually thinned upon reduction and extended along the rolling direction. The IMC layer was a hard-brittle phase with the largest hardness and low plasticity. Its thickness was basically unchanged during the deformation, and it was fragmented during the coordinated deformation. The fragmentation partially extended along the rolling direction of the Al layer and the substrate. When the IMC layer was fragmented and developed large gap, the outer Al layer filled in the gap. The underlying mechanism is shown in Figure 7.

**Figure 8:**
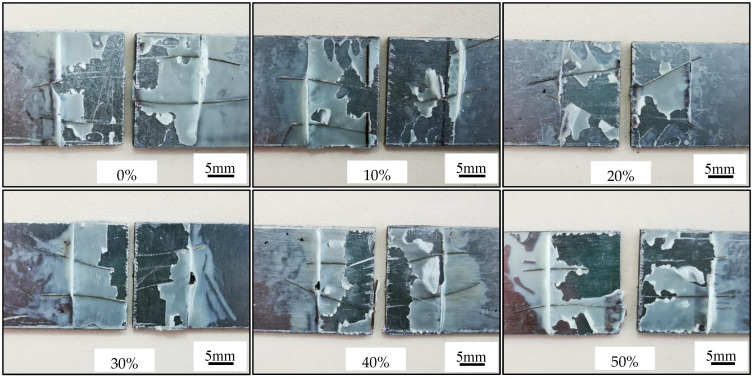
Lap shear strength testing of AS60/60 coating at different reduction ratios.

**Figure 9:**
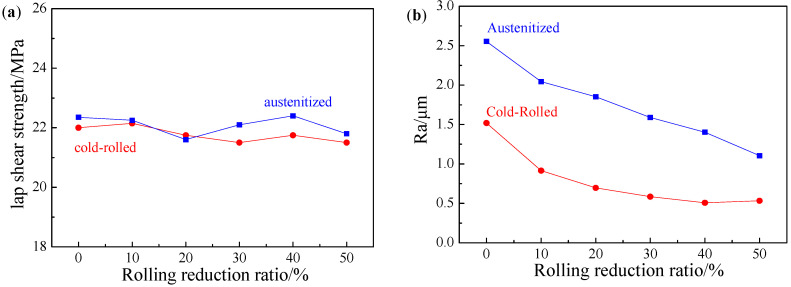
(**a**) Lap shear strength of AS60/60 coating at different reduction ratios; (**b**) surface roughness (Ra) of AS60/60 coating at different reduction ratios.

The adhesive force tests and results of AS60/60 coatings are illustrated in Figure 8 and Figure 9a, respectively. The failure type was adhesive failure (AF) for the cold-rolled coatings at different reduction ratios. Namely, visible failure occurred at the interface between the adhesive agent and the adhered substance, and the coatings were not destroyed. It was indicated the adhesion force between the adhesive agent and the coating was smaller than the cohesion force of the coating or the cohesion force of the adhesive agent.

The lap shear strength was 22.00 ± 1.25 MPa in the hot-dipped AS60/60 coatings, and was 22.15 ± 1.25, 21.75 ± 1.25, 21.50 ± 1.35, 21.75 ± 1.20, 21.50 ± 1.40 MPa, respectively in the cold-rolled AS60/60 coatings from 10% to 50% reduction ratio, indicating the results are basically consistent. The reason is that, in spite of rapid diffusion channels formed during cold rolling, the cohesive forces of the coating and the adhesive agent are both larger than the adhesive force between them, and this adhesive force corresponds to the shear strength, so this strength will not change with the cold rolling reduction ratio.

The surface roughness of the AS60/60 coatings at different cold rolling reduction ratios was shown in Figure 9b. The surface roughness of the AS60/60 coatings decreased with the increase of cold rolling reduction ratio, and was stabilized at the cold rolling reduction ratio of 40% and above. The hardness of the Al layer in the hot-dipped coatings and the cold-rolled roller surface was about 70 HV (Ra = 1.5 μm) and 810 HV (Ra = 0.3 μm), respectively, so the surface roughness of the rollers would be transferred to the Al layer during the cold rolling. As the cold rolling reduction ratio increased, the Al layer was gradually thinned, and its duplicated roller surface roughness was intensified, which resulted in a decrease in surface roughness of coatings. When the reduction ratio was 40% and above, the surface roughness of coatings was stable. This was because the Al layer was squeezed into the cracks of the IMC layer, which reduced the duplication of the surface roughness of the rollers and made the surface roughness of AS60/60 coatings stable.

### 3.2. Microstructure, Thickness and Adhesive Force of Austenitized Coating

**Figure 10:**
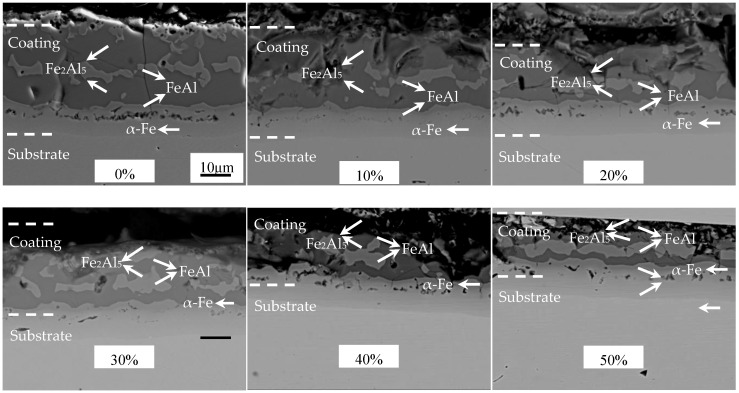
EPMA micrographs of AS60/60 coating at different reduction ratios at temperature of austenization (TAUS) = 930 °C for 5 min.

The cold-rolled coatings after austenitization were displayed in Figure 10. The structures of the coatings were significantly changed after austenitization. The austenitized AS60/60 coatings were structurally continuous and stratified with clear boundaries. The coatings at different positions were analyzed with EDS (Table 3). After the coatings cold-rolled at different reduction ratios were austenitized, the coating structures were consistent. Namely, the structures of the austenitized coatings did not change with the cold rolling reduction ratio. Each coating, from the surface to the steel substrate, consisted of a Fe_2_Al_5_ layer, a FeAl layer, a Fe_2_Al_5_ layer, a FeAl layer, and a α-Fe layer [17]. The Fe_2_Al_5_ layer, the FeAl layer, and the Fe_2_Al_5_ layer are called Al-Fe layers, and the FeAl layer and the α-Fe layer are called diffusion layer. The formation of the α-Fe layer is mainly attributed to the ability of element Al to stabilize the body centered cubic (BCC) structure of Fe.

**Figure 11:**
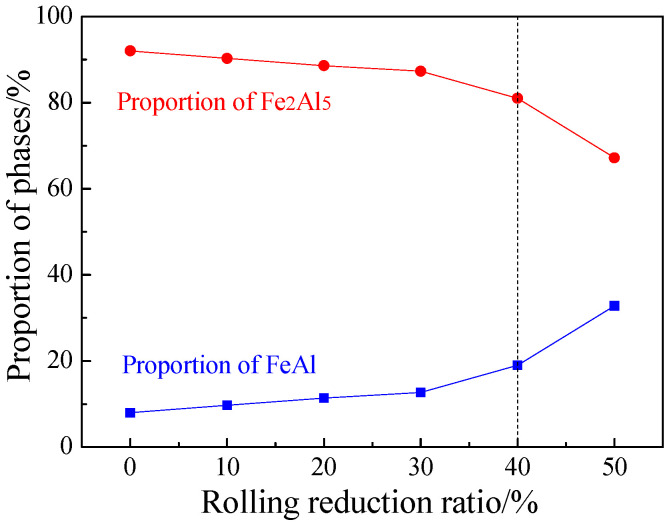
Proportion of Al-Fe layer phases at different reduction ratios at TAUS = 930 °C for 5 min.

The area proportions of the Fe_2_Al_5_ phase and the FeAl phase in the Al-Fe layer after austenitization at different cold rolling reduction ratios were shown in Figure 11. As the cold rolling reduction ratio increased, the proportion of Fe_2_Al_5_ gradually decreased, but the proportion of FeAl rose. As the cold rolling reduction ratio rose, the coatings became thinner, improving the heating efficiency of PHS during austenitization, so the coatings melted more quickly, and there was sufficient time for the Fe element in the substrate to diffuse to the coatings. In the meantime, the thinning of coatings shortened the Fe diffusion distance to the coatings, which decreased the proportion of Al-rich Fe_2_Al_5_ and increased the proportion of Fe-rich FeAl. The cold-rolled coatings at reduction ratio ≥40% contained rapid diffusion channels, which enlarged the direct contact area between the steel substrate and the Al layer, so Fe can more directly and quickly diffuse to the Al layer, promoting the transition from Al-rich Fe_2_Al_5_ to Fe-rich FeAl and further increasing the proportion of FeAl.

**Figure 12:**
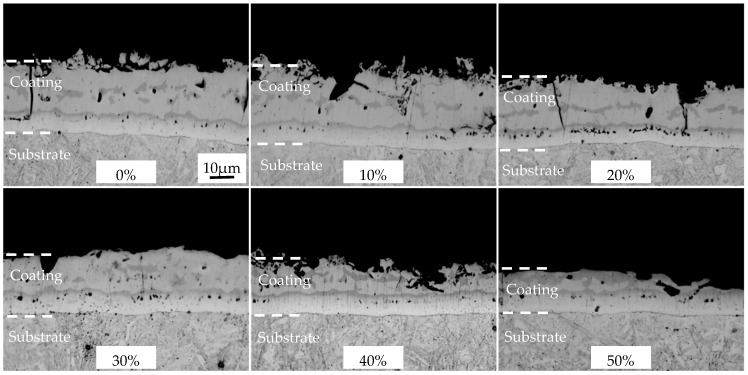
OM micrographs of AS60/60 coating at different reduction ratios at TAUS = 930 °C for 5 min.

**Figure 13:**
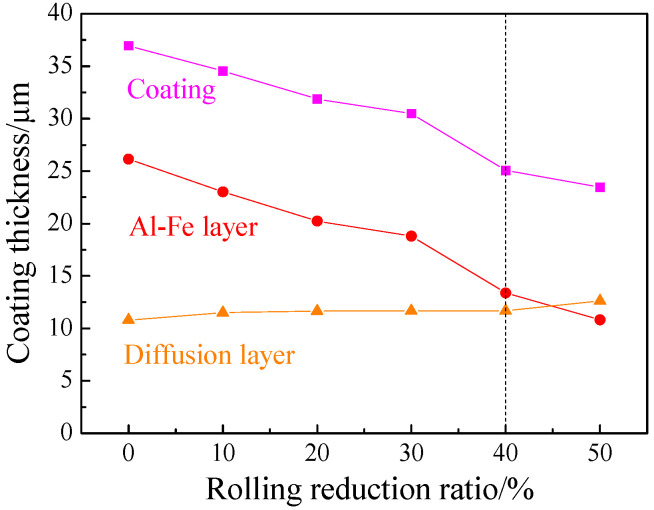
Thickness of AS60/60 coating at different reduction ratios at TAUS = 930 °C for 5 min.

The structures and thickness of the austenitized coatings at different cold rolling reduction ratios were shown in Figure 12 and Figure 13. The structural morphology of the coatings was basically unchanged with the increase of cold rolling reduction ratio, and was composed of an Al-Fe layer and a diffusion layer, and the coatings were continuous on the whole. The austenitized coatings without reduction were 36.94 μm thick, which included the Al-Fe layer thickness of 26.14 μm and the diffusion layer thickness of 10.80 μm. As the reduction ratio increased, the coating thickness was shortened in a nearly linear way, and the thickness shortening rate was accelerated when the reduction ratio was ≥40%. The changing trend of the Al-Fe layer thickness was consistent with that of coating thickness, but the diffusion layer thickness was enlarged from 10.8 to 12.6 μm. The changing trends of coating thickness and Al-Fe layer thickness were mainly attributed to the changing trend of the cold-rolled coating layer thickness. The thickness of the diffusion layer increased at small amplitude and was basically stable. On the one hand, the thinning of the cold-rolled layer improved the heating efficiency, accelerating the Al diffusion rate to the steel substrate. On the other hand, the chemical potential of Al induced by the thinning of cold-rolled coatings decreased, weakening the diffusion driving force of Al to the steel substrate. These two factors were balanced, so the diffusion layer did not change significantly.

**Figure 14:**
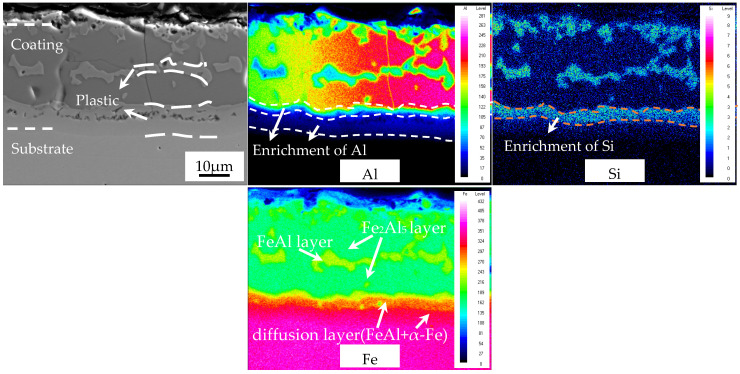
EDS map scan result of interface between the AS60/60 coating and the steel substrate at 0% reduction ratio at TAUS = 930 °C for 5 min.

When the AS60/60 coatings deformed without reduction were austenitized, the map-scanned zone clearly consisted of a Fe_2_Al_5_ layer, a FeAl layer, a Fe_2_Al_5_ layer, a diffusion layer, and the steel substrate (Figure 14). In the Fe_2_Al_5_ layer, the Al and Fe concentrations were relatively stable, the Al concentration was large, but the Si concentration was low. In the FeAl layer, the Al, Fe, and Si concentrations were relatively stable, and the Al and Fe concentrations were larger than the Si concentration. In the diffusion layer, only Al and Si were enriched near the Fe_2_Al_5_ layer, which corresponds to the FeAl phase (2.2 μm thick). Al was enriched near the steel substrate, which corresponded to the α-Fe phase (which also proved Al element accounted for the formation of the α-Fe phase). In the steel substrate, the Fe concentration rapidly increased and maximized, but the Al and Si concentrations rapidly declined. The distribution of element concentrations is consistent with the structural analysis of austenitized coatings above.

**Figure 15:**
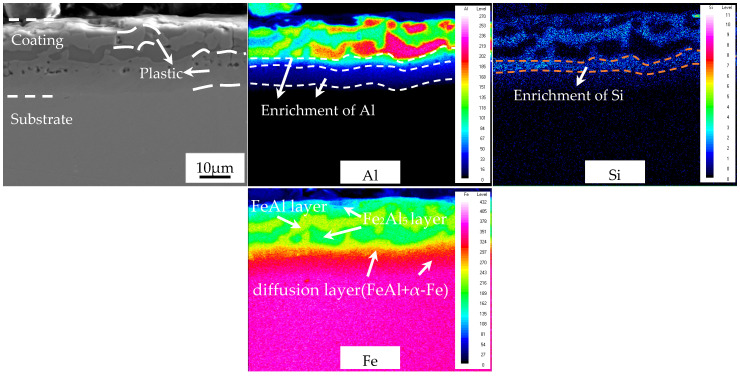
EDS map scan result of interface between the AS60/60 coating and the steel substrate at 50% reduction ratio at TAUS = 930 °C for 5 min.

The overall distributing rules of Fe, Al, and Si in the cold-rolled coatings at 50% reduction ratio after austenitization are consistent with the cold-rolled coatings without reduction ratio (Figure 15). In other words, the cold rolling reduction ratio did not affect the Fe, Al, and Si diffusion or the phase formation in the AS60/60 coatings after austenitization, which is consistent with the EDS results.

**Figure 16:**
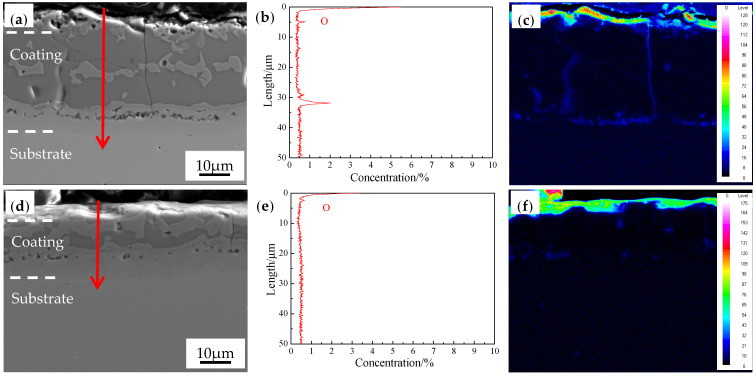
EPMA of interface between the AS60/60 coating and the steel substrate at TAUS = 930 °C for 5 min: (**a**,**d**) AS60/60 coatings at 0% and 50% reduction ratio, respectively, and (**b**,**e**) the corresponding EDS line scan results obtained along the red arrow; (**c**,**f**) EDS map scan results of oxygen element at 0% and 50% reduction ratio, respectively.

To analyze the antioxidation abilities of the coatings at varying thickness, we subjected the austenitized coatings to line scan and map scan of oxygen element (Figure 16). At the cold rolling reduction ratios 0% and 50%, oxygen was concentrated on the coating surface, and the thicknesses of the coatings were 2.7 and 3.6 μm, respectively, which are basically consistent. Oxygen was enriched in the internal cracks and pores of the coatings, which was mainly because the cracks became the routes for oxygen to get into the coatings, and the densities of cracks and pores after cold rolling at 50% reduction ratio were smaller than those after treatment at 0% reduction ratio. At the cold rolling reduction ratio of 50%, the oxygen enrichment in the coatings decreased, which improved the antioxidation ability.

**Figure 17:**
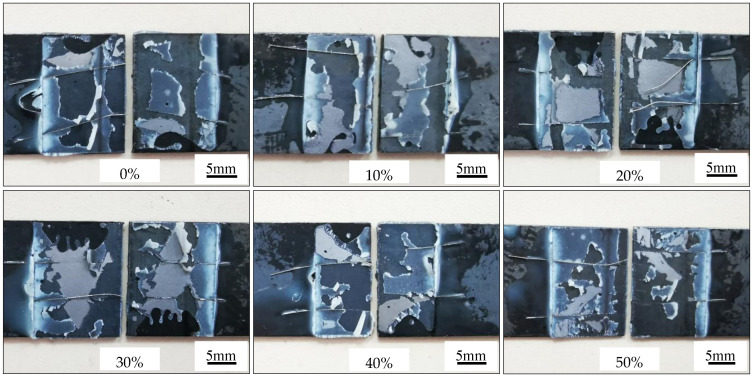
Lap shear strength testing of AS60/60 coating at different reduction ratios at TAUS = 930 °C for 5 min.

The adhesive force tests and results of the coatings at different cold rolling reduction ratios after austenitization are illustrated in Figure 17 and Figure 9, respectively. The failure type was AF after the cold-rolled coatings at different reduction ratios were austenitized. Namely, visible failure occurred at the interface between the adhesive agent and the adhered substance, and the coatings were not destroyed. It was indicated the adhesion force between the adhesive agent and the coating was smaller than the cohesion force of the coating or of the adhesive agent.

The lap shear strength was 22.35 ± 1.20 MPa in the original AS60/60 coatings, and was 22.25 ± 1.40, 21.60 ± 1.15, 22.10 ± 1.15, 22.40 ± 1.30, 21.80 ± 1.40 MPa respectively in the AS60/60 coatings from 10% to 50% reduction ratios (Figure 9a). The failure type and shear strength were consistent between the two original and reduced coatings, indicating the strength did not change with the variation of cold rolling reduction ratio.

The surface roughness of the austenitized AS60/60 coatings at different cold rolling reduction ratios was shown in Figure 9b. The surface roughness of the austenitized coatings was larger than that of the cold-rolled coatings, but decreased as the cold rolling reduction ratio rose. In the austenitized coatings, high-density intermetallic compounds were formed, and the coating volume was contracted, leading to an increase in the surface roughness of the coatings [30]. As the cold rolling reduction ratio rose, the coating thickness decreased, and the volume decreasing rate of the corresponding austenitized coatings declined. As a result, as the cold rolling reduction ratio increased, the surface roughness of the austenitized coatings decreased.

### 3.3. Bending Toughness of Al-Si Coated PHS

**Figure 18:**
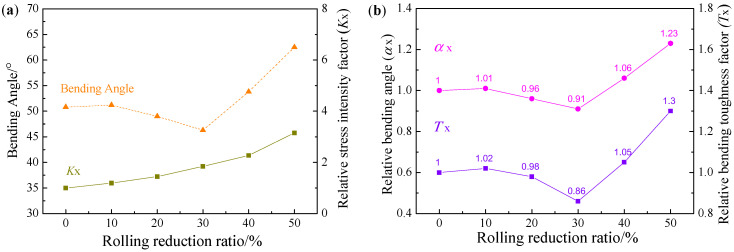
(**a**) Bending angle and Kx at different reduction ratios at TAUS = 930 °C for 5 min; (**b**) α_x_ and T_x_ at different reduction ratios at TAUS = 930 °C for 5 min.

The results of bending angle of PHS at different reduction ratios after austenitization were shown in Figure 18a. The bending angle gradually decreased with the rise of cold rolling reduction ratio in the range ≤30%, but rapidly increased at the reduction ratio ≥40%. The bending angle at the reduction ratio of 50% was enlarged by 23% from that at the reduction ratio of 0%. During the bending deformation, the diffusion layer cannot prevent crack expansion in the coatings. Based on three-point bending tests, we suppose there was only one crack at the center of the top bending surface. Thus, for the coating crack that ran through the Al-Fe layer and the diffusion layer, its tip stress intensity factor K_SIF_ was calculated by Equation (1), where F is the applied force, L is the distance between two supports, W and t are the width and thickness of a specimen, respectively, and a is the crack length. Since the cracks run through the Al-Fe layer and the diffusion layer, a is the total thickness of the coating [28]. For PHS specimens cold-rolled at different reduction ratios, K_SIF_ is only decided by the thickness t and crack length a. The K_SIF,x_ (x is the cold rolling reduction ratio) divided by the K_SIF,0%_ of the original blank is named the relative stress intensity factor K_x_. As the cold rolling reduction ratio rose, K_SIF,x_ gradually increased or namely K_x_ rose, because the effect of coating thickness reduction was smaller than that due to specimen thickness reduction. If the bending toughness was only related to K_SIF_, then K_SIF,x_ increased as the cold rolling reduction ratio rose, leading to a larger local shear deformation in the martensite substrate. On this basis, the bending angle shall gradually decrease, but actually, the bending angle suddenly increased when the reduction ratio was ≥40%. This difference indicates K_SIF_ alone cannot fully explain the bending toughness.

(1) $$K_{\mathrm{SIF}}=\frac{3FL}{2Wt^{2}}\sqrt{\pi a}$$

Cracks always start from the coating surface, so the bending toughness is more sensitive to coating cracks [28]. The Fe_2_Al_5_ phase had larger hardness (HV 900–1000 vs. HV 580–650) and lower fracture toughness (1.2 vs. 26 MPa·m^1/2^) than the FeAl phase [17]. As the cold rolling reduction ratio rose, the brittle Fe_2_Al_5_ phase was more converted to the tough FeAl phase after austenitization, leading to a gradual increase in the proportion of FeAl in the Al-Fe layer, which decreased the generation and expansion of coating cracks during the bending process, alleviated the local shear deformation caused by the martensite substrate, and improved the bending resilience. Thus, the bending toughness is affected by both K_SIF_ and FeAl proportion. The P_FeAl,x_ (the proportion of FeAl at reduction ratio x) divided by the P_FeAl,0%_ of the original blank is named the relative FeAl proportion P_x_. The bending toughness is correlated negatively with K_SIF_ and positively with FeAl proportion, so we define the relative bending toughness factor T_x_ (Equation (2)). The α_bending,x_ (bending angle at reduction ratio x) divided by the α_bending,0%_ of the original blank is named the relative bending angle α_x_. The relative bending toughness factor is highly consistent with the relative bending angle, with errors less than 6% (Figure 18b). The relative bending toughness factor can well reflect the changing rule of the bending angle of hot stamping steel, and fully reveals the effects of K_SIF_ and FeAl proportion on the bending angle.

(2) $$T_{x}=\frac{P_{x}}{K_{x}}$$

## 4. Conclusions

(1) As the cold rolling reduction ratio increased, the overall continuity of AS60/60 coatings was unchanged. When the cold rolling reduction ratio was ≥40%, the Al layer filled in the cracks of the IMC layer, forming rapid diffusion channels. The formation mechanism of rapid diffusion channels is that during cold rolling, the Al layer and the steel substrate are deformed in terms of thickness reduction, but the IMC layer is deformed in terms of fragmentation. When the IMC layer is fragmented and cracked to form large gap, the Al layer with the lowest hardness will fill in the gap. The coating thickness was shortened with the increase of cold rolling reduction ratio. The rapid diffusion channels and coating thickness thinning did not affect the adhesive force of coatings, 21.50–22.15 MPa, and the failure type of coatings belonged to adhesive failure (AF). The surface roughness of the AS60/60 coatings decreased with the increase of cold rolling reduction ratio, and was stabilized at the reduction ratio of 40% and above;
(2) After the coatings cold-rolled at different reduction ratios were austenitized, the coatings were structurally consistent and composed of an Al-Fe layer and a diffusion layer. As the reduction ratio increased, the coating thickness was shortened and diffusion layer thickness was basically stable, while the adhesive force of coatings was 21.60–22.40 MPa and the failure type of the coatings was still adhesive failure (AF). Simultaneously, the surface oxygen distribution thickness of the austenitized coatings was 2.7–3.6 μm, the cracks and pores of austenitized coatings decreased, which decreased the oxygen enrichment in the coatings and further improved the antioxidation ability. The surface roughness of the austenitized coatings was larger than that of the cold-rolled coatings, but decreased as the reduction ratio rose; and
(3) When the cold rolling reduction ratio was ≥40%, owing to the presence of rapid diffusion channels, the proportion of brittle Fe_2_Al_5_ rapidly decreased and the proportion of FeAl phase rose quickly after the austenitization, leading to a rapid increment in the bending toughness in PHS. When the cold rolling reduction ratio was 50%, the bending angle was improved by 23%. The relative bending toughness factor can well reflect the changing rule of bending angle of Al-Si-coated PHS.

## Figures and Tables

**Table 1:** Chemical composition of the experimental steel (mass %).

C	Si	Mn	P	S	Cr	Mo	Ti	Al	B	Cu
0.218	0.238	1.102	0.015	0.0006	0.168	0.008	0.034	0.022	0.0028	0.034

**Table 2:** Energy dispersive spectroscope (EDS) of AS60/60 coating in the hot-dipped condition (at. %).

Al	Si	Fe	Phase
97.18	2.51	0.31	Al
85.54	7.35	7.11	Al + τ_6_
67.27	12.25	20.48	τ_5_

**Table 3:** EDS of AS60/60 coating at TAUS = 930 °C for 5 min (at.%).

Al	Si	Fe	Phase
68.74	1.44	29.82	Fe_2_Al_5_
44.29	13.55	42.16	FeAl
10.14	2.64	87.22	α-Fe

## Data Availability

The data presented in this study are available on request from the corresponding author.
